# DLProv: a suite of provenance services for deep learning workflow analyses

**DOI:** 10.7717/peerj-cs.2985

**Published:** 2025-07-01

**Authors:** Débora Pina, Liliane Kunstmann, Adriane Chapman, Daniel de Oliveira, Marta Mattoso

**Affiliations:** 1 COPPE Institute, Universidade Federal do Rio de Janeiro, Rio de Janeiro, Brazil; 2 School of Electronics and Computer Science, University of Southampton, Southampton, United Kingdom; 3 Institute of Computing, Universidade Federal Fluminense, Niterói, Brazil

**Keywords:** Traceability, Provenance data, W3C PROV, Provenance graph, Deep learning

## Abstract

Deep learning (DL) workflows consist of multiple interdependent and repetitive steps, including data preparation, model training, evaluation, and deployment. Each step involves decisions impacting the final model’s performance, interpretability, and applicability. These models rely on data, preprocessing operations, and configuration, underscoring the need for mechanisms to ease the analysis throughout the entire life cycle—from model generation and selection to deployment. Moreover, ensuring trust, reproducibility, and transparency becomes important as DL models transition into production environments. Traceability across the steps of the DL workflow is essential to address these challenges. However, existing traceability solutions often present limitations. Many fail to integrate the steps of the DL workflow, focusing on either data preparation or model training. Additionally, they frequently rely on proprietary formats to represent traceability data and rarely produce a provenance document that can accompany the model into production. To bridge these gaps, we present DLProv, a suite of provenance services designed to ensure end-to-end traceability across DL workflows. DLProv supports structured query language (SQL)-based querying during training and generates provenance graphs that capture data preparation steps, model training, and evaluation. These provenance graphs comply with the PROV *de facto* standard, ensuring interoperability across different environments. One of the key strengths of DLProv lies in its framework-agnostic architecture. The suite’s services can be invoked independently of the DL framework, enabling integration across several training and deployment workflows. Furthermore, DLProv includes specialized instances designed for specific DL frameworks, such as Keras and physics-informed neural networks (PINNs), offering adaptability to a wide range of applications. We evaluated DLProv using well-established datasets, including Modified National Institute of Standards and Technology (MNIST) and Canadian Institute for Advanced Research (CIFAR)-100. These datasets were chosen to illustrate the suite’s capability to capture and manage provenance data across tasks of varying complexity, from basic image classification to more complex DL workflows. Additionally, we evaluated DLProv within a handwritten transcription workflow, further showcasing its flexibility. Across all these use cases, DLProv showed its ability to ease SQL-based queries during model training while maintaining framework independence. An important aspect of our evaluation was measuring the overhead introduced by integrating DLProv into DL workflows. The results showed a maximum overhead of 1.4% in execution time, highlighting the suite’s minimal impact on DL workflow performance. For comparative analysis, we benchmarked this overhead against MLflow, further reinforcing DLProv’s suitability for real-world DL applications.

## Introduction

Deep learning (DL) is a subset of machine learning (ML) that supports decision-making processes by focusing on training computational models composed of multiple layers of nonlinear processing units. These layers enable the learning of hierarchical data representations, which result in a DL model (LeCun, Bengio & Hinton, 2015). The aforementioned hierarchical learning capabilities depend on leveraging large, preprocessed datasets. Once trained, a DL model can identify patterns in new datasets. As a result, DL has become a transformative technology, advancing fields such as natural language processing (NLP), computer vision (CV), and speech recognition (SR) (Chai et al., 2021).

DL models are generated by preparing datasets to be trained and validated by a deep neural network (DNN) architecture. A DL model generation typically involves workflow steps such as data preprocessing, model training, hyperparameter tuning, and model validation, all of which produce artifacts and metadata that contribute to the model’s final form. Due to the variety of alternative configurations for each workflow step, several candidate DL models are usually generated. Finally, a model selection stage identifies the most suitable DL model for further deployment. This involves comparing candidates using data such as performance metrics.

After being selected for deployment, a DL model transitions into production, where it becomes a part of real-world systems and applications. As discussed by Souza et al. (2024), the complexity of DL models with their application in critical decision-making requires trust in model predictions. Provenance tracking in DL workflows has emerged as an essential support for this trust and interpretability (Ferreira da Silva et al., 2024). Providing comprehensive provenance tracking for DL workflows requires capturing and relating metadata from all the workflow steps (Souza et al., 2022; Pina et al., 2024, 2023, 2025). Despite the existing initiatives in the literature, provenance tracking in DL workflows is an open, yet important, problem (Ferreira da Silva et al., 2024; Leo et al., 2024).

Current DL frameworks often employ proprietary traceability representations, creating an ecosystem that makes interpretability difficult. We consider that provenance capture for traceability should not be tightly coupled to the DL framework (Pina et al., 2024). Existing solutions for monitoring and analyzing DL models typically concentrate on providing analyses through metadata management without traceability. While some claim to support traceability, they often lack the representation of typical relationships, thus restricting the ability to trace derivation paths across the DL workflow stages. With the diverse landscape of DL frameworks and execution environments, DL scientists should be free to use different frameworks independently for each DL workflow stage. Unlike traditional software systems, DL workflows often run in environments ranging from cloud-based platforms to high-performance computing (HPC) clusters, with each environment imposing its requirements and limitations. This scenario contributes to the challenge of DL workflow provenance tracking and calls for a solution that flows along different frameworks and platforms to generate a DL provenance graph.

We did not find a solution that generates traceability support that integrates the DL development stages and delivers a provenance graph document to follow the DL model in production workflows. DLProv (Pina, 2025a) addresses this gap in the literature and current solutions by presenting a suite of provenance services. DLProv services produce traceability documents as provenance graphs that integrate the DL model traces. The contributions of DLProv support are that it is World Wide Web Consortium (W3C) PROV (Moreau & Groth, 2013) compliant, and its services can be invoked independently from the DL frameworks chosen to follow the DL model stages. DLProv has captured provenance in notebooks, clusters, cloud, and HPC machines. It has also supported DL workflows in frameworks like TensorFlow (https://www.tensorflow.org), PyTorch (https://pytorch.org/), and DeepXDE (Lu et al., 2021). Even when new DL models appear like physics-informed neural networks (PINNs) (Toscano et al., 2025) or surrogate generative adversarial networks (GANs), DLProv has shown its benefits under the same services and provenance model. Our goal with DLProv is to enhance analytical capabilities during the development and selection of DL models and contribute to aspects of trust, interpretability, and reproducibility in models in production.

This work is organized as follows: “Background: Challenges in Traceability of DL Workflows” provides the background, detailing foundational concepts relevant to the proposed suite. “Related Work” discusses related work, highlighting existing approaches and their limitations. “Suite of Provenance Services” introduces the proposed DLProv suite, describing its architecture and functionalities, while “DLProv Specializations” presents DLProv specializations for capturing and analyzing provenance in DL workflows. “DLProv Suite Evaluation” presents the experiments conducted to validate the suite and their results and analysis. Finally, “Conclusion and Future Work” concludes the article, summarizing the findings and outlining future directions.

## Background: challenges in traceability of DL workflows

This section introduces important concepts of this article. It introduces concepts involved in the DL workflow, providing an overview of the steps. Then, we highlight the impact of human actions in DL workflows, exploring the pivotal role of human intervention. Finally, the concepts of traceability and their role in DL workflows are presented.

### An overview of DL workflow components

**Figure 1:**
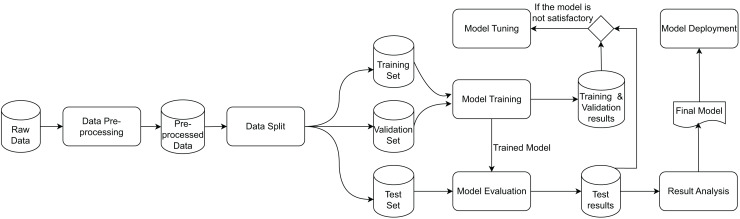
Data transformations and artifacts involved in the DL workflow (Pina et al., 2023).

DL workflows are both data-centric and model-centric, as they produce a DL model based on input raw data through a data transformation flow (Schlegel & Sattler, 2023a). While Schlegel & Sattler (2023a) explicitly represents the relationships between processes, they do not depict the data. Therefore, Fig. 1 presents a simplified view of the DL workflow from a dataflow perspective, inspired by representations such as Miao et al. (2017), Gharibi et al. (2021), Shankar & Parameswaran (2022), Polyzotis et al. (2018), Xin et al. (2018). The workflow consists of data transformations represented as rounded rectangles and datasets represented by cylinders. The workflow begins with data preparation, an important step involving a series of preprocessing operations. These operations can impact a DL model’s performance, motivating data scientists to experiment with diverse combinations of operations, often referred to as preprocessing pipelines, that address transformations at structural and syntactical levels. Once preprocessing is complete, the prepared data is split into training and testing datasets to facilitate the evaluation of the DL model. This split can occur either before or after the data preparation phase, depending on the specific operations involved.

The DL workflow contains feedback loops among the different data transformations, which are characteristics of an experimentation process. Therefore, it often involves additional iterative refinements and going back to previous steps based on performance metrics. For instance, when training error remains high, strategies such as increasing model capacity, extending training duration, or changing the architecture may be explored. Similarly, high validation errors can be addressed by incorporating more data, adjusting regularization, or reconsidering the model design. During model evaluation, performance issues on test data may prompt efforts to align training data with test data, collect additional data, or revisit architectural choices. These iterations are implicitly depicted in the model tuning transformation.

### The role of traceability in the DL workflow

Human intervention adds a critical layer of expertise to the DL workflow. Data scientists, with their domain knowledge, actively engage in the analysis of DL workflow results. They interpret the findings, tune the DL workflow configurations, and ensure that the selected model not only excels in terms of quantitative metrics but also aligns with the specific needs of the task at hand. To enable such informed decision-making, integrating data transformations, configurations, and results within a DL workflow is important.

Decision-making benefits of traceability to understand relationships within the workflow. Provenance is a natural and standard solution for traceability (Pina et al., 2024). Unfortunately, many metadata-based approaches do not provide traceable relationships. These approaches often lack relational structures in their data models, treating metadata as isolated attributes rather than connected entities. This limitation increases the burden on data scientists, who must manually infer relationships that should be explicitly supported by the system.

For instance, consider a workflow designed to predict whether a person earns more than $50 K annually using the Adult Census dataset (Becker & Kohavi, 1996). If an analysis aims to trace the prediction for a specific person back to the data preparation steps, such as encoding their “Education” level, *e.g*., Bachelor’s, Master’s, or Doctorate, it is necessary to establish a relationship between preprocessing and DL model training activities. In solutions based on metadata, the connection between preprocessing steps and DL training steps may be lost, due to the lack of explicit relationships, making it difficult to understand the transformations applied to a given input (data for a specific person). On the other hand, solutions that associate preprocessing activities with the training activity provide a trace from a DL model back to the specific preprocessing that shaped the preprocessed data. Such traceability is also valuable in deployment scenarios. If the DL model presents unexpected prediction values or performance issues arise, the provenance traceability is a reliable means to investigate inconsistencies.

**Figure 2:**
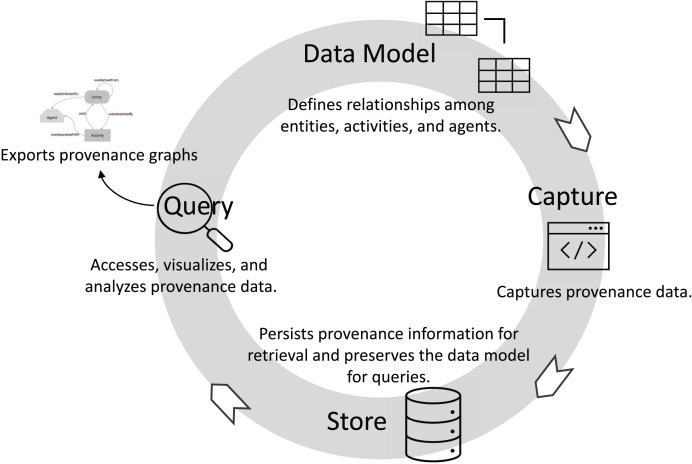
Tracking entities, activities, and relationships throughout the DL life cycle.

Promoting traceability consists of several key phases: data model, capture, store, and query (visualization/analysis), as presented in Fig. 2. The first phase, data model, involves defining a provenance data model, typically following an established standard like W3C PROV. In this phase, the relationships among agents, activities, and entities are specified, allowing for the abstract representation of provenance data. The data model relationships should define how entities are generated, used, and derived to enable traceability. In addition, accountability can be established by the relationships between agents and activities. During the capture phase, provenance information is captured in real-time as activities are executed. For example, in a DL workflow, the training data (an entity) and the transformation steps (activities) are tracked, capturing how data evolves and which models (entities) are generated by specific training activities. The *wasGeneratedBy* and *used* relationships are commonly recorded during this phase, documenting the data transformations and the sequence of operations. Captured provenance data is then stored in the store phase, often within a provenance-aware database or graph system. Proper storage ensures data integrity and supports data retrieval for analysis. Finally, the query phase enables data scientists to analyze provenance information through querying and visualization tools. By tracing relationships and gaining insights into workflow processes, data scientists can identify bottlenecks, optimize configurations, and ensure compliance with governance and accountability standards.

When provenance is integrated directly within ML frameworks, it allows for the automatic capture of provenance data during workflow execution, reducing the overhead associated with manual intervention. However, this tight coupling can lead to challenges in flexibility, as the provenance capture capabilities may be limited by the framework’s architecture and functionalities. Additionally, changes or updates to the framework may disrupt provenance tracking, potentially impacting reproducibility and trust in the captured data. On the other hand, using external provenance systems allows for greater flexibility and can facilitate the incorporation of provenance across arbitrary execution frameworks. This approach can be beneficial in heterogeneous environments where multiple ML frameworks are in use. However, it often requires additional effort to implement and maintain, as it may involve instrumentation to ensure that relevant provenance information is captured. This can introduce complexity and the potential for errors if not managed carefully.

### Exporting provenance graphs for production

In DL workflows, traceability is important to enhance analytical capabilities during DL model development, selection, and integration into production environments (Mora-Cantallops et al., 2021). The two main approaches for exporting traceability information are log files and provenance graphs. This section details the strengths and limitations of each approach, comparing their roles in DL workflow analysis.

Log files are easy to implement and can be used to capture workflow activities and represent the workflow results as entities, but are limited in their ability to structure complex relationships (Barringer et al., 2010). While log files can provide basic analytical capabilities and a transparent record of actions, their structure can fall short when tracking data transformations and traceability (Yao et al., 2016), which is important for reliable reproducibility and trust in production. Provenance graphs represent the workflow structure, capturing entities (data, models, parameters), activities, and their relationships (Missier, Belhajjame & Cheney, 2013; Herschel, Diestelkämper & Lahmar, 2017). Typically stored in a database, provenance graphs provide detailed workflow traceability, enabling fine-grained analytical capabilities during model development and selection. This traceability enhances reproducibility, interpretability, and trust in production environments (Mora-Cantallops et al., 2021), where model auditability and transparent decision-making are essential.

For storage, log files are typically saved as plain files on an operating system, such as a disk. Provenance graphs, on the other hand, are stored in established databases designed for structured data storage, such as relational or graph databases. When it comes to concurrency and consistency, log files are susceptible to issues such as log corruption during simultaneous writes, whereas provenance graphs leverage database systems that provide built-in concurrency control, minimizing corruption risks.

Interpretation of log files depends heavily on user-defined formats and requires knowledge of the specific log structure, whereas the standardized structure of provenance graphs simplifies interpretation, with inherent relationships encoded within the data. Traceability is another area where differences are apparent. Log files often require post-processing to establish traceability, like manual setups, such as sorting by timestamps, to establish event order and traceability. Provenance graphs, in contrast, capture entity relationships, including dependencies, facilitating traceability. Lastly, querying capabilities differ substantially. Log files offer limited querying functionality, making complex analyses challenging. Provenance graphs, however, support complex relational or graph-based queries, simplifying sophisticated analysis tasks.

## Related work

DL workflows generate vast amounts of data and metadata, yet many existing tools fail to capture the relationships and dependencies needed to understand and reproduce the process of training DL models. Traceability is often limited by simplistic metadata logs or systems that provide only fragmented views of workflow steps, neglecting the relationships between activities, data, and results. Without robust traceability, it becomes challenging to reconstruct the models’ derivation trace, validate the results’ reproducibility, or analyze the impact of specific workflow choices. This lack of provenance also undermines the ability to perform advanced queries and ensure transparency. We did not find any solution that delivers a provenance graph document to follow the DL model in production. This lack of provenance documents of the DL model in production limits the ability to establish trust in the deployed model.

Mora-Cantallops et al. (2021) review the state of the art, as of 2021, on models and tools for ensuring trustworthiness in AI. They analyze key transparency requirements through traceability, drawing on the EU Commission’s Ethics Guidelines for Trustworthy AI (https://digital-strategy.ec.europa.eu/en/library/ethics-guidelines-trustworthy-ai). Their study emphasizes the role of provenance in supporting accountability and interpretability in AI systems. Additionally, they highlight the importance of adopting standards to ensure compliance with ethical and regulatory frameworks. This analysis aligns with our research, as we also emphasize the role of provenance in enhancing transparency and interoperability, as well as supporting analyses in AI systems, particularly within DL workflows.

Current popular solutions like MLflow (https://mlflow.org/) (Zaharia et al., 2018; Chen et al., 2020) and Weights & Biases (WandB) (https://wandb.ai/site) provide user-friendly interfaces for managing ML workflows. MLflow is an open-source framework that enables users to track experiments by embedding tracking commands within Python scripts. It logs parameters, metrics, and artifacts, with storage options ranging from local files to SQLAlchemy-compatible databases or remote tracking servers. Despite its widespread adoption, MLflow lacks native support for provenance traces and relationship-driven representations. As a result, it does not generate provenance documents to accompany DL models when they are deployed. WandB also offers a solution for managing ML models, from the experimentation phase to model production. However, despite allowing the definition of some relationships between artifacts, WandB requires scripting for a more in-depth analysis to traverse the resulting graph, in addition to requiring prior knowledge of the graph depth. During hands-on experience with WandB, we also identified some issues with respect to its network requirements. When WandB is executed, it requires access to external IP addresses. In supercomputer environments like Santos Dumont (https://sdumont.lncc.br/), the compute nodes typically do not allow external access, making it necessary to configure the firewall to allow such connections. However, in practice, this configuration is not always feasible due to security policies or technical restrictions, limiting the usability of WandB in such HPC facilities.

These solutions are grounded in metadata-based log systems, which provide a simplistic model for recording workflow steps. These models typically suffer from limitations such as the absence of relationships between activities, representing only attributes of individual steps without a holistic view. This lack of a relationship-driven data model restricts trace representations, impacting the ability to perform robust queries and analyses.

Addressing some of these gaps, MLflow2PROV (Schlegel & Sattler, 2023b) extends MLflow by generating PROV-compliant provenance graphs based on information extracted from code repositories and MLflow. While this approach enhances traceability, its reliance on MLflow’s limited captured data results in incomplete provenance support. For example, MLflow2PROV struggles to document preprocessing steps applied to data before model training when such steps are not explicitly recorded in Git or MLflow logs. Additionally, its provenance graph can become overloaded with Git-related details, making it challenging to perform queries that require a clear representation of workflow activities and their relationships.

The Braid Provenance Engine (Braid-DB) (Pruyne, Wozniak & Foster, 2022; Wozniak et al., 2022) captures provenance of data and steps in ML workflows, with a focus on producing data products that are findable, accessible, interoperable, and reusable (FAIR). It manages ML model versions, enabling traceability back to raw data, including scientific datasets. Braid-DB captures provenance data at the file and version levels rather than at the record level. Although it is not fully compliant with the W3C PROV recommendation, it covers core PROV concepts and supports analytical queries.

DPDS (Chapman et al., 2022, 2024; Gregori et al., 2024), which stands for Data Provenance for Data Science, is an approach designed to foster explainability, reproducibility, and trust in ML models by automatically tracking granular provenance related to raw data preprocessing operations that precede model training. DPDS provenance is compliant with W3C PROV and ensures that the complete data preparation step is captured in detail, providing a transparent record of how the data was handled.

MLtrace (https://mltrace.readthedocs.io) (Shankar & Parameswaran, 2022) is a data management system that offers debugging for deployed ML workflows through assisted detection, diagnosis, and reaction to ML-related bugs and errors. MLtrace allows for the automatic logging of inputs, outputs, and metadata associated with execution, and it has an interface for data scientists to ask arbitrary *post-hoc* queries about their pipelines.

ModelDB (Vartak et al., 2016; Vartak & Madden, 2018) is an open-source system to manage ML models, tracking model metadata through the whole ML workflow, *e.g*., parameters of preprocessing operations, hyperparameters, *etc*. While ModelDB offers traceability, it does not follow the W3C PROV recommendation for provenance data representation. Additionally, ModelDB is tightly integrated with specific ML frameworks, which can restrict its adoption across different ML frameworks.

ModelHub (Miao et al., 2016, 2017) is a system designed for managing DL models, offering a model versioning system that allows for storing, querying, and tracking different model versions. It also features a domain-specific language, which acts as an abstraction layer for searching through the model space, along with a hosted service to store, explore, and share developed models. Since it primarily focuses on the model itself, ModelHub does not capture information about other DL workflow steps, such as data preparation, limiting its scope for end-to-end traceability.

ModelKB (Gharibi et al., 2021) is a Python library focused on managing DL models with automatic metadata extraction. ModelKB stores metadata regarding the model architecture, weights, and configurations, which allows for reproducibility, querying, visualization, and comparison of experiments. The primary goal of ModelKB is to offer model management with minimal disruption to the data scientist’s workflow by using callbacks to capture metadata. However, it does not follow recommendations for provenance data representation.

ProvLake (https://ibm.biz/provlake) (Souza et al., 2022) provides provenance services through lightweight tracking that can be easily integrated into workflow code, such as scripts. This tracking can be applied at each step of the workflow and subsequently integrated into a data lake. By using a universal identifier, ProvLake captures relationships within the data lake and offers data modeling for all workflow steps in compliance with W3C PROV. ProvLake has been used in ML workflows and offers traceability of the ML workflow. Although it operates independently of specific frameworks, ProvLake requires consistent use throughout all steps of the workflow to ensure comprehensive tracking.

Runway (Tsay et al., 2018) is a prototype tool for tracking ML experiments that organizes metadata about ML models, similar to tools like MLflow and WandB. It offers visualization capabilities to facilitate the exploration of relationships between hyperparameters and metrics. While Runway is intended to be framework-agnostic, it currently provides integration only with Python scripts through a Python SDK.

Vamsa (Namaki et al., 2020) is a system that extracts provenance from Python ML scripts without requiring changes to the data scientists’ source code. It essentially analyses scripts to determine which columns in a dataset have been used to train a certain ML model, automatically recording the relationships between data sources and models at a coarse-grained level.

Amazon SageMaker (https://aws.amazon.com/sagemaker/) (Nigenda et al., 2022) provides resources for model building, training, deployment, and metadata tracking. SageMaker provides traceability to some extent through its built-in capabilities for tracking model metadata, such as training configurations, metrics, and model artifacts. However, this traceability is largely limited to its internal environment, with no support for exporting detailed provenance traces to external systems. SageMaker relies on proprietary formats for logging and metadata management. In addition, SageMaker is tightly coupled with Amazon Web Services (AWS), making it less adaptable for use in diverse execution frameworks. This dependency on AWS infrastructure reduces its flexibility for integrating with external tools or workflows beyond its ecosystem.

Table 1 assesses some of the existing solutions based on the following criteria: independence, Traceability, DL Provenance Graph, Provenance Representation, and Provenance Graph in Production. Independence highlights whether a solution supports arbitrary execution frameworks. Traceability assesses whether a solution captures the necessary information so that provenance traces can be generated, encompassing all steps of DL workflows. DL Provenance Graph evaluates whether a solution produces a provenance graph derived from the captured traceability. Provenance Representation shows whether the solution adheres to any standards for provenance representation. Provenance Graph in Production indicates if a solution supports exporting the generated provenance graph for use in production.

**Table 1:** Assessment of solutions according to Independence, Traceability, DL Provenance Graph, Provenance Representation, and Provenance Graph in Production.

System	Independence	Traceability	DL provenance graph	Provenance representation	Provenance graph in production
Braid-DB	Yes	Yes	No	N/A	No
DPDS	Yes	No	No	PROV	No
MLflow	Yes	No	No	N/A	No
MLflow2PROV	Yes	No	No	PROV	Yes
Mltrace	Yes	Yes	No	N/A	No
ModelDB	No	Yes	No	PMML	No
ModelKB	Yes	No	No	N/A	No
ModelHub	Yes	No	No	NNEF/ONNX	No
ProvLake	Yes	Yes	Yes	PROV	No
Runway	Yes	No	No	N/A	No
SageMaker	No	No	No	N/A	No
Vamsa	Yes	No	No	N/A	No
WandB	Yes	Yes	No	N/A	No
DLProv	Yes	Yes	Yes	PROV	Yes

Table 1 shows that most existing solutions fail to provide provenance support across the steps of DL workflows. While some systems like Braid-DB, Mltrace, and WandB offer traceability, few solutions offer a complete DL life cycle provenance graph or the ability to export the provenance graphs into production. The adoption of provenance representation standards is limited, with only a few tools, such as DPDS, MLflow2PROV, and ProvLake, leveraging established standards like PROV. Independence is provided by more solutions, with more than half of the solutions supporting arbitrary execution frameworks. For instance, MLflow and WandB are highly versatile, compatible with a wide range of ML frameworks such as TensorFlow, PyTorch, and scikit-learn, while Vamsa facilitates provenance capture in Python scripts. DPDS also supports Python scripts, but data should be in the form of Pandas dataframes.

## Suite of provenance services

Generating a provenance graph is a natural solution for traceability. Therefore, we designed the DLProv suite (https://github.com/dbpina/dlprov) (Pina, 2025a) to integrate provenance services into DL workflows, thereby supporting detailed analyses and enhancing both reproducibility and trust in DL models. DLProv builds on DfAnalyzer (Silva et al., 2020; Silva, 2018) core provenance components to address traceability challenges in DL workflows. DLProv suite encompasses all traceability key phases (Fig. 2): data model, capture, store, query (visualization/analysis), and contributes with an export service that delivers provenance documents to assist DL models in production. These services can be invoked in DL workflow scripts or embedded in frameworks, as was done in Keras (Pina, 2025b), or specialized for scientific DL like PINNProv (de Oliveira, 2025). Its compliance with W3C PROV has shown the interoperability of DLProv with other tools that capture provenance at different DL workflow stages, including data preparation.

**Figure 3:**
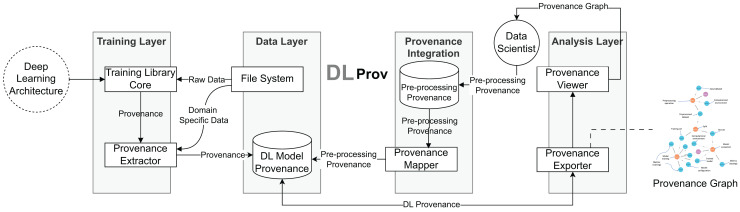
DLProv suite architecture (Pina et al., 2025).

The DLProv suite architecture was introduced in Pina et al. (2021), with an updated version briefly presented in Pina et al. (2025). It consists of four service layers, as shown in Fig. 3. In the Training Layer, the Provenance Extractor captures both prospective and retrospective provenance from DL workflows developed using DL frameworks such as TensorFlow or PyTorch. Prospective provenance (p-prov) refers to the specification that outlines how data should be generated or processed, while retrospective provenance (r-prov) relates to the data associated with each step during execution, such as parameter values (Freire et al., 2008). During model training, all captured provenance data is sent to the Data Layer for persistent storage in the DL Model Provenance database. The Provenance Integration Layer then connects the training-related provenance with the provenance data from data preparation. Finally, the Analysis Layer offers resources for exporting the captured provenance in standard document formats.

### Provenance data model

A provenance graph is a structured representation that models the dataflow, processes, and relationships within a system. It provides an account of how data is generated, transformed, and used by capturing interactions between entities (data or artifacts), activities (processes or data transformations), and agents (people or systems). In this directed graph, nodes represent the core elements—entities, activities, and agents, while directed edges represent relationships such as *used*, *wasGeneratedBy*, and *wasDerivedFrom* (Huynh et al., 2018; Belhajjame et al., 2013).

Consider a scenario where a DL model is being trained. The resulting provenance graph for this process would have entities and activities as nodes. Entities would be the raw dataset and intermediate datasets produced during preprocessing, hyperparameters, and metrics. Activities would be data preparation steps, DL model training, and validation processes. The edges would be the relationships between the activities and entities. *Used* edges would connect activities to the entities they use. For instance, an edge may link a training activity to the preprocessed dataset it uses. *WasGeneratedBy* edges would indicate the generation of new entities by specific activities. A model training activity, for example, would be linked to the trained model it produces.

**Figure 4:**
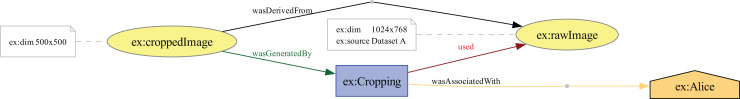
A W3C PROV representation of an image cropping activity transforming a raw image entity into a cropped image entity.

The data preparation can also be represented in the provenance graph, as in the example shown in Fig. 4. For instance, consider a dataset of flower images where each image contains elements like surrounding grass or background. A cropping operation could isolate only the flower in the image. Suppose the original raw image is 1,024 × 768 pixels, and the flower is centered within a 500 × 500-pixel square. The cropping operation would extract this 500 × 500 region and discard the rest, creating a processed image that highlights the flower and minimizes noise in the dataset. In W3C PROV terms, the raw image is represented as an entity, named *ex:rawImage*, with metadata specifying its dimensions (1,024 × 768 pixels) and source (Dataset A). This image is connected to a *ex:Cropping* activity through the *used* relationship, indicating that it served as input for the operation. The activity can include metadata about its start and end times, as well as specific parameters used to define the rectangular area to be cropped from the raw image. The resulting cropped image is another entity, named *ex:croppedImage*, with updated dimensions (500 × 500 pixels). This entity is linked to the cropping activity through the *wasGeneratedBy* relationship, indicating that the cropping activity produced this new image. Additionally, the cropped image is linked back to the original raw image through the *wasDerivedFrom* relationship, establishing that the cropped image originates from the raw image. To ensure complete traceability, the cropped image can also be connected to the aforementioned training activity, illustrating the data’s flow from preparation to DL model training. The provenance graph also includes an agent, representing the data scientist who performed the operation, identified as *ex:Alice*; This agent is connected to the cropping activity with the *wasAssociatedWith* relationships, indicating responsibility for executing the operation.

**Figure 5:**
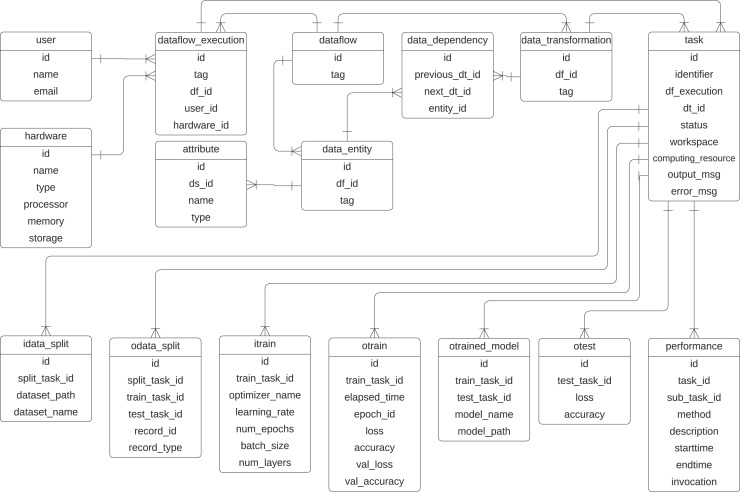
Provenance data representation for DL workflows. Extended from Pina et al. (2023).

To represent the provenance information captured in DL workflows, we propose a specialized provenance data model derived from the domain-agnostic PROV-DM (Belhajjame et al., 2013). Given that most ML workflows follow a common structure, typically involving data preparation, data splitting, model training, and testing, we adapt PROV-DM to explicitly incorporate these elements. This specialization ensures that the provenance data model captures details of DL workflows while maintaining compatibility with PROV-DM. Figure 5 presents the UML class diagram of the proposed provenance data model. The initial version of this model was introduced in Pina et al. (2021) and later refined in Pina et al. (2023), adding classes that highlight the relationships between data transformations within the DL workflow. This provenance data model is tailored to capture training-specific data from DL experiments while integrating them into the preprocessing transformations performed on the data.

The classes related to the dataflow specification are inherited from the PROV-Df model (Silva et al., 2016), which are *dataflow*, *data_transformation*, *data_entity*, *data_dependency*, and *attribute*. These classes represent prospective provenance. At its core, the *dataflow* class represents the workflow, identified by an id and a descriptive tag. The execution of a specific instance of dataflow is managed by the *dataflow_execution* class. Within a dataflow, individual steps, *i.e*., activities, are defined as *data_transformation*, such as *train*.

Data or entities used or generated in the workflow are captured by the *data_entity* class. In previous articles, we have used this class with the name *data_set* as any collection of data elements to be consumed or produced during an activity. However, to avoid confusion with a dataset that serves as input for training a DL model, we employ the term *data_entity* in the current provenance data model. The *performance* class monitors task performance, recording attributes such as method descriptions, start and end times, and invocation details. Relationships between transformations are tracked using the *data_dependency* class, which links transformations through their *previous_dt_id* and *next_dt_id*, along with the associated data entities.

To represent retrospective provenance, the provenance model contains the class *task*, which represents the instances of activities that are executed, which allows for recursive queries that trace back the execution. The input of the training process is captured in the *itrain* class, which includes parameters such as the optimizer name, learning rate, number of epochs, batch size, and number of layers. The training outcomes are represented by the *otrain* class, which stores metrics like loss, accuracy, validation loss, and validation accuracy for each epoch. The resulting trained models are managed by the *otrained_model* class, which includes identifiers for the associated training task, model name, file path, and related test task. Testing processes are tracked in the *otest* class, which records test accuracy and loss. Data splits used in training and testing are handled by the *idata_split* and *odata_split* classes, with attributes to identify the dataset paths, names, and task identifiers for training and testing. The schema also supports the integration of hardware resources through the hardware class, while user information is maintained in the user class.

With these classes, the data scientist can discover which preprocessing methods were applied to the data to train/evaluate a model. The prefix *i* refers to the input parameters of a task, and the prefix *o* in the classes refers to the output parameter values. This model is designed to be extensible, allowing the incorporation of additional elements, such as hyperparameters, metrics, and data transformations, as needed for more detailed analysis. For example, the activity *ex:Cropping* and the resulting entity *ex:croppedImage*, shown in Fig. 4, could be integrated into the provenance data model to be captured.

### Provenance capture

DLProv operates independently of the DL framework, as its services are invoked through script instrumentation. This approach enables it to capture parameters and metrics, such as shown in the provenance model in Fig. 5, and to track dependencies between activities within the workflow. The data scientist defines which domain data, model configurations, and model metrics will be captured and where they should be captured. With this instrumentation, the Provenance Extractor can access the data during training. For instance, Listing 1 illustrates a fragment of the instrumentation for a simple workflow. In this example, the workflow takes a preprocessed dataset, splits it into training and testing subsets, and then uses the training set to train a DL model. A dependency, such as *dependency = t1*, means that activity *t2* (*e.g*., model training) can only start after activity *t1* (*e.g*., data splitting) has produced the required output. For simplicity, the inputs and outputs of activity *t1* are omitted in the figure.

**Listing 1:** Fragment of DLProv instrumentation showing activity dependencies in a simple DL workflow.

1 dataflow_tag = “example”
2 df = Dataflow(dataflow_tag, predefined=True)
3 df.save()
4 exec_tag = dataflow_tag + “-” + str(datetime.now())
5 t1 = Task(1, dataflow_tag, exec_tag, “SplitData”, dependency = t0)
6 class DLProvCallback(Callback):
7 def on_epoch_end(self, epoch, logs=None):
8 tf2_output = DataSet(“oTrain”, [
9 Element([timestamp, elapsed_time, loss, accuracy,
10 val_loss, val_accuracy, epoch])])
11 t2.add_dataset(tf2_output)
12 t2.save()
13 t2 = Task(2, dataflow_tag, exec_tag, “Train”, dependency = t1)
14 callbacks = DLProvCallback(t2)
15 tf2_input = DataSet(“iTrain”, [Element([optimizer_name,
16 learning_rate, epochs, batch_size, num_layers])])
17 t2.add_dataset(tf2_input)
18 t2.begin()
19 model.compile(optimizer=opt,
20 loss=‘sparse_categorical_crossentropy’,
21 metrics=[‘accuracy’])
22 model.fit(x_train, y_train, epochs=epochs, validation_split=0.2,
23 callbacks=callbacks)
24 tf2_output_model = DataSet(“oTrainedModel”, [Element([trained_model,
25 trained_model_path])])
26 t2.add_dataset(tf2_output_model)
27 t2.end()

Consider the cropping scenario. The workflow starts with a raw image entity that undergoes a cropping operation. Once cropping is applied to all raw images in the dataset, the resulting cropped images are used in subsequent steps, such as splitting the data into training and testing sets, followed by training a DL architecture. Here, the splitting activity is identified as *t1*, and the training activity as *t2*, with *t2* explicitly dependent on the completion of *t1*. This dependency ensures that the training process cannot begin until the split images are available.

Intermediate steps, such as additional preprocessing operations applied to the cropped images before training (*e.g*., data augmentation or normalization), would also depend on the output of the cropping operation. This creates a chain of dependencies, where each activity is linked to the successful completion of the preceding step, ensuring the workflow’s traceability.

### Provenance store

Once captured, the provenance data is sent asynchronously with the DL execution to the Data Layer. The DLProv suite uses a database referred to as DL Model Provenance in Fig. 3, currently instantiated with MonetDB (https://www.monetdb.org/), a columnar database optimized for analytical workloads, as its provenance storage solution. This database is designed to manage and query the provenance data generated throughout DL workflows, adhering to the provenance data model shown in Fig. 5.

During the training of a DL model, provenance data is asynchronously sent to the DL Model Provenance database, ensuring minimal interference with the training process. This allows scientists to execute SQL queries on the data while the DL model is still being trained. Even after the training phase finishes, the provenance data remains stored in the database, enabling continued analysis and querying to support reproducibility and performance evaluation.

In some cases, provenance capture for different stages of a DL workflow may be handled by separate solutions, for example, one solution capturing provenance during data preparation and another during DL model training. To address this scenario, assuming both solutions adhere to the W3C PROV recommendation, the Provenance Integration Layer ensures integration of the provenance information collected by these solutions. To allow the integration between these data models of two provenance databases, the Provenance Mapper obtains the identifier for each record or image and inserts this identifier in the DL Model Provenance database, along with the type of data transformation that will use that record.

### Provenance queries

During the development and selection of DL models, scientists rely on queries to analyze metadata captured during training. This metadata includes details about training epochs, parameters, and the performance of candidate models, such as in Fig. 5. By querying this database, scientists can make informed decisions about selecting the “best” candidate for deployment. Once a DL model transitions into production, queries play an important role in ensuring reproducibility and verifying consistency. For instance, scientists may use queries to confirm whether the preprocessing steps applied during model development were replicated in the production environment. These checks are vital for detecting discrepancies that could compromise the reliability of the deployed model.

The Analysis Layer provides data scientists with analytical capabilities, enabling them to perform queries during training and explore provenance data in greater depth after training. While many analyses can be conducted directly through SQL queries, some require a provenance graph to better explore relationships and dependencies within the workflow. One of the advantages of using the W3C PROV is the ability to generate predefined provenance document representations, such as PROV-N, which can later be converted and analyzed in graph database management systems (DBMS).

DLProv supports both p-prov and r-prov, which provide a relationship from the specification of the DL workflow to all alternative executions of this workflow. In DLProv, the identifier df_tag is associated with the p-prov, representing the overall workflow specification. For each instance of this workflow execution, r-prov is generated with a df_exec identifier. Queries may use the relationship from df_tag to df_exec to analyze and filter candidate DL models. With the *Provenance Exporter*, scientists can create a provenance document in PROV-N format. Scientists can export all provenance graphs associated with the workflow or a specific subset of these workflow executions through DBMS queries that follow joins between the df_tag and df_exec. Both identifiers, df_tag and df_exec, are predefined in the code and can be retrieved from MonetDB. To query the relationships, the DLProv suite includes functionality for ingesting the provenance document into Neo4j (https://neo4j.com), using PROV Database Connector (https://github.com/DLR-SC/prov-db-connector). Once ingested, the provenance data can be queried using the Cypher language.

Table 2, adapted from Pina et al. (2024), outlines a set of queries used during two key stages of the workflow: *(i)* the training and selection of DL models, and *(ii)* their deployment and use in production. These queries, adapted from the literature and the Provenance Challenges (https://openprovenance.org/provenance-challenge/WebHome.html), vary in complexity—some focus on single DL workflow activities, such as DL model training. In contrast, others trace the derivation path of artifacts across multiple stages. In the Workflow Stage column, we distinguish between Development and Deployment stages. A query classified under Development can be performed during the generation and selection of DL models, leveraging data from multiple executions and configurations. Queries marked as Deployment focus exclusively on the provenance data of the deployed DL model, providing insights into its behavior and derivation trace in the production environment. If a data scientist needs to analyze additional executions or configurations beyond the deployed model, the workflow is assumed to revert to the Development stage for further exploration. This distinction clarifies the scope and applicability of each query within the DL workflow.

**Table 2:** Examples of typical provenance queries in DL workflows.

Id	Query	Workflow stage	Source
Q1	Find all trained models with a specific value for the learning rate.	Development	Provenance challenge 1
Q2	What was the epoch’s average processing time of the model training?	Development, Deployment	Souza et al. (2022)
Q3	What is the hyperparameter configuration used to train the model with the highest average training accuracy?	Development	Provenance challenge 1
Q4	What is the hyperparameter configuration used to train the model with the highest test accuracy?	Development	Provenance challenge 1
Q5	For a given experiment, which data contributed to the run with the highest test accuracy?	Development	Provenance challenge 1
Q6	Given a training set, what are the values for hyperparameters and evaluation measure associated with the trained model with the least loss?	Development, Deployment	Souza et al. (2022)
Q7	Which hyperparameters were used in this model?	Development, Deployment	Schelter et al. (2017)
Q8	What was the computational environment used to train a given model?	Development, Deployment	Provenance challenge 4
Q9	Who was responsible for training a given model?	Development, Deployment	Provenance challenge 4
Q10	Find the process that led to a given model (*i.e*. model M)/everything that caused model M to produce these results.	Development, Deployment	Provenance challenge 1
Q11	Which data was used to train this model?	Development, Deployment	Namaki et al. (2020)
Q12	What feature transformations have been applied to the data?	Development, Deployment	Schelter et al. (2018)

When training a DL model with several configurations, queries can provide insights into the process and outcomes of the experiments. These queries enable comparison and selection of the most suitable model for deployment by analyzing training configurations, performance metrics, and other information. They support the evaluation of multiple training runs, helping to identify configurations that yield the best results or optimize specific criteria. For instance, Q1 helps filter the experiments. By determining the average time per epoch during training, Q2 can provide insights into the computational performance of different configurations. Q3 enables the data scientist to evaluate which configurations are more likely to generalize well to unseen data, offering a starting point for further tuning or testing. Q4 focuses on selecting the model most likely to perform well in real-world scenarios, where the test accuracy is a proxy for deployment reliability. Q5 traces back the data that played a role in achieving the best test accuracy, providing a clear link between the input data and model performance. With Q6, data scientists can analyze the relationship between different hyperparameter configurations and DL model performance.

Once a model is deployed, the provenance document becomes an important resource for querying information about its performance and behavior. Post-deployment queries can focus on the reproducibility of the model’s performance under different conditions or the impact of any changes made during deployment. These queries provide insights into how the model interacts with new data and whether adjustments to the deployment environment are needed. They also play an important role in model monitoring, troubleshooting, and auditing, ensuring that the model operates as expected. For instance, Q7 can reveal the exact hyperparameters used during training, which is important for understanding how different configurations (*e.g*., learning rate or batch size) influenced the model’s performance. This knowledge can inform future retraining or tuning efforts. Understanding the computational environment where the model was trained (*e.g*., GPU specifications, memory, OS, or specific software versions), as in Q8, is important for reproducing the training setup, ensuring consistency across different production environments, and troubleshooting potential environment-related issues that might arise during deployment. Q9 identifies the individual or team responsible for training the model, offering accountability and context when revisiting decisions made during training, such as choices related to hyperparameters or data preprocessing. Q10 provides a detailed provenance trail of all steps leading to the creation of a model. This is important in deployment scenarios where accountability is required, ensuring that all details during model training and validation are transparent and documented. Q10 also ensures that the original processes are well-documented, facilitating efficient reproducibility of results. Q11 and Q12 are particularly beneficial as they provide information about the datasets used to train the model. These queries ensure that the data preparation process is traceable and that the provenance document includes detailed information about any data transformations. In a production setting, they help verify that the model was trained on the intended data and serve as an audit trail for evaluating model performance or addressing any data-related issues. Finally, knowing the feature transformations applied to the training data, as queried in Q12, is important for understanding how preprocessing affected the model’s performance. For example, if feature scaling, encoding, or dimensionality reduction was used, tracking these steps ensures that future changes in preprocessing do not unintentionally alter the model’s behavior. Q12 also helps identify discrepancies between the transformations applied to the data during DL model training and those used during deployment, ensuring consistency in preprocessing steps and minimizing the risk of performance degradation due to mismatched transformations.

Given a dataset that underwent multiple preprocessing steps, such as normalization and augmentation, a data scientist might ask: *How did normalization impact model performance across different training runs?* Answering this requires retrieving models trained on normalized and non-normalized data, comparing their accuracy, and preserving the full trace from raw data to final metrics. To support such a query, the provenance must capture relationships between model accuracy and the processes that generated it. Specifically, each model’s accuracy is derived from training or evaluation activities (*wasGeneratedBy* relationship in W3C PROV graph), which in turn used specific datasets (*used* relationship in the provenance graph). These datasets, whether train, validation, or test, were produced through a data splitting activity that operated on preprocessed data. This preprocessed dataset resulted from a sequence of preprocessing activities, such as normalization and augmentation, which must be explicitly linked through relationships like *used*, *wasGeneratedBy*, and *wasDerivedFrom*, all tracing back to the original raw data. Thus, to fully answer Q12, it is important to establish and maintain all these relationships, ensuring a traceable path from raw data to model evaluation.

Consider an example where a DL model is trained on the Framingham Heart Study (FHS) dataset to predict heart disease risk (Pina et al., 2023), the training data primarily consists of individuals aged 30–70, with most in their 50s and 60s. When deployed in a healthcare application monitoring younger individuals (20–30 s), the model may fail to generalize due to the underrepresentation of this age group in training. To investigate this issue, scientists can examine the provenance trace with the deployed model. These relationships can help trace preprocessing steps, understand training data distribution, and track key transformations. Beyond detecting distribution shifts, provenance traces can also be used for model comparison. For instance, scientists can query historical provenance data to identify past models trained on more diverse age groups or alternative preprocessing strategies. Comparing these models’ performance in production can help determine whether retraining with additional data or adjusted preprocessing techniques would improve accuracy.

## DLProv specializations

The DLProv suite includes specialized instances tailored to specific use cases of DL workflows. These specializations are distinguished by their integration with the *Training Service Layer*, using components *Training Library Core* and *Provenance Extractor*. Initially, DLProv was considered to support analyses over DL model training and evaluation in arbitrary execution frameworks (Pina et al., 2021). This section introduces three key instances: DLProv for Keras, DLProv for PINNs, and DLProv for the DL life cycle.

### DLProv for Keras

KerasProv (https://github.com/dbpina/keras-prov) (Pina, 2025b; Pina et al., 2021) was developed to show that DLProv services can be explicitly invoked from DL scripts or can be embedded into different DL frameworks. KerasProv components’ architecture acts as provenance plugins to the software that executes the DL workflows, in this case, Keras API (https://keras.io/). KerasProv invokes DLProv provenance services within the functionalities of the Keras API. KerasProv is a library with a Python interface that provides provenance data to Keras DL applications. The core idea behind KerasProv’s architecture is to preserve the original Keras structure while enabling the online registration of model configurations, evaluation metrics, and their relationships as provenance data. This ensures minimal interference with the scientist’s workflow while maintaining provenance documentation.

For KerasProv, the *Provenance Extractor* is embedded directly within the Keras library, eliminating the need for manual instrumentation by the data scientist. Instead, the data scientist selects predefined provenance options, domain data, and hyperparameters to be captured. The *Provenance Extractor* then automatically extracts and stores the values of the hyperparameters used in each training iteration. The code snippet in Listing 2 shows how provenance data can be captured for a Keras model.

**Listing 2:** Code snippet showing how to use DLProv for Keras. Adapted from Pina et al. (2021).

1 hyperparameter_values = {“OPTIMIZER_NAME”: True,
2 “LEARNING_RATE”: True,
3 “DECAY”: False,
4 “MOMENTUM”: False,
5 “NUM_EPOCHS”: True,
6 “BATCH_SIZE”: True,
7 “NUM_LAYERS”: True}
8
9 model.provenance(dataflow_tag=“KerasProv-example”,
10 adaptation=True,
11 hyperparameters = hyperparameter_values)

In this example, the *hyperparameter_values* dictionary defines which hyperparameters are captured during training. Each key in the dictionary corresponds to the name of a hyperparameter (*e.g*., OPTIMIZER_NAME, LEARNING_RATE), while the boolean values indicate whether the respective hyperparameter should be included in the provenance data. The model.provenance() function is then invoked to capture this information, using the dataflow_tag parameter to assign a unique identifier to the model’s dataflow. This minimal addition to the code is sufficient to enable provenance capture, as illustrated in Listing 2, without requiring further instrumentation.

This specialization shows how DLProv can be embedded into DL frameworks to avoid manual instrumentation. However, the current implementation is coupled with a specific version of Keras, which limits its compatibility. Nevertheless, this approach highlights how the Keras API and other frameworks could adopt provenance capture as a built-in plugin, making such capabilities readily available for all users in a compatible representation.

### DLProv for PINNs

Since DLProv provides flexibility in terms of arbitrary execution frameworks, we extended the provenance data model and, consequently, *Provenance Extractor*, to capture provenance for PINNs, which have specific model configurations and model metrics and are often trained using specific frameworks like DeepXDE (Lu et al., 2021) and SciANN (Haghighat & Juanes, 2021).

To capture provenance when using DeepXDE, a specialized library for PINNs, it is necessary to create a class that inherits from the *deepxde.callbacks.Callback* class. This approach allows PINNProv (de Oliveira, 2025; de Oliveira et al., 2023), an instance of DLProv for PINNs, to leverage DeepXDE methods, which are executed during key steps in the training process of the PINN model. The code example in Listing 3 shows how manual instrumentation is carried out, following the structure outlined in Listing 1. Specifically, the code shows how to capture provenance at key moments during training: when the model training begins (*on_train_begin*), when it ends (*on_train_end*), and after each epoch (*on_epoch_end*).

**Listing 3:** Code snippet showing how to use DLProv for PINNs. Adapted from de Oliveira et al. (2023).

1 class PINNProv(deepxde.callbacks.Callback):
2 def __init__(self):
3 (…)
4 df = Dataflow(dataflow_tag, [‘OPTIMIZER_NAME’,
5 ‘LEARNING_RATE’, ‘EPOCHS’, ‘BATCH_SIZE’, ‘LAYERS’,
6 ‘WEIGHT_LR’, ‘WEIGHT_LB’, ‘WEIGHT_LD’],
7 [‘epoch’, ‘time_elapsed’, ‘LOSS’, ‘LR_train’, ‘LB_train’,
8 ‘LD_train’, ‘Q_train_error’, ‘U_train_error’])
9 df.save()
10 def on_train_begin(self):
11 (…)
12 t1 = Task(1, dataflow_tag, exec_tag, “Train”)
13 tf1_input = DataSet(“itrain”, [Element([opt_name, l_rate,
14 epoch, batch, layers_list, weight_lr, weight_lb, weight_ld)])
15 t1.add_dataset(tf1_input)
16 t1.begin()
17 def on_epoch_end(self):
18 tf1_output = DataSet(“otrain”, [Element([epoch, elapsed_time,
19 loss_value, lr_value, lb_value, ld_value, err_q_train,
20 err_u_train])])
21 t1.add_dataset(tf1_output)
22 t1.save()
23 model.train(…, callbacks=[PINNProv()])

In addition to traditional hyperparameters and metrics, PINNs have specific metrics unique to their architecture. These include loss components such as *WEIGHT_LR*, *WEIGHT_LB*, and *WEIGHT_LD*. The ability to capture these specialized metrics, along with any other relevant data, ensures that the full range of training details can be recorded as provenance. With the use of *callback*, PINNProv can be used with DeepXDE for any PINN specification and any of its *back-ends*.

### DLProv for DL life cycle

The DLProv suite includes an instance as an integration mechanism for provenance data that enables the capture and merge of information across different stages of a DL workflow. Specifically, the Provenance Integration Layer serves as a bridge that facilitates the integration of provenance data, whether it originates from preprocessing, model training, or other steps within the DL workflow. While the Provenance Integration Layer is designed to be adaptable and extendable to accommodate different provenance capture solutions, its current implementation focuses on integrating data from two key sources: preprocessing steps, as captured by Chapman et al. (2020), and DL model training and selection, as captured by DLProv.

In the preprocessing step, provenance data is collected by a dedicated module that tracks transformations and modifications made to the raw data. This could include normalization, feature extraction, and data augmentation. The provenance information gathered in this step includes not only the transformations applied to the data but also metadata such as the parameters used in each operation, the timestamp of when the transformations occurred, and the resulting data artifacts. By capturing this information, a solution ensures that the data preparation step is fully traceable, essential for reproducibility and transparency in DL workflows. Once the data is prepared, the training process begins. A data scientist can leverage DLProv to capture provenance throughout the DL model’s training.

The Provenance Integration Layer consolidates this diverse provenance data into a unified structure that can be queried, visualized, and analyzed. This integration is crucial for supporting complex analyses, such as identifying correlations between data transformations and model performance, detecting issues related to data leakage, or ensuring that the same preprocessing steps used during training are consistently applied when the model is deployed in production.

To enable the integration of provenance data models from two provenance capture tools, the first step is to obtain a unique identifier for each data record (*e.g*., record_id), which can be retrieved by querying the Data Preparation Provenance database, responsible for storing the provenance data for the preprocessing operations. Once the record_id is obtained, it is inserted into the DL Model Provenance database (in MonetDB), along with the type of data transformation associated with that record (*i.e*., whether the record will be used for training or evaluation of a DL model). Since these records play a crucial role in the model development and selection process of the DL workflow, and MonetDB serves as the provenance database during this stage, the record_id attribute must be present in both provenance models to establish a foreign key relationship between the two databases.

## DLProv suite evaluation

In this section, we discuss the experiments conducted in this article to evaluate the proposed suite. Three experiments were carried out. The first two aimed to show DLProv’s provenance capture over data preparation, DL model training, and evaluation, on arbitrary platforms such as TensorFlow and PyTorch, benefiting from the same provenance representation. These experiments also show that the DLProv suite can be used with popular script parallelization tools like Parsl (https://parsl.readthedocs.io/en/stable/index.html), which served as a workflow orchestrator by distributing tasks across two GPUs to maximize resource utilization since our focus is on DL workflows that run on HPC systems and can be parallelized using different type of libraries. The third experiment focuses on a handwritten transcription workflow, aiming to show DLProv’s capability to integrate new data for capture. The provenance data model can be extended to capture new data through manual instrumentation. Specifically, this experiment highlights DLProv’s ability to document preprocessing activities tailored to the workflow’s domain. These experiments were designed to show DLProv’s capability to provide traceability of the DL workflow for analysis during the training of DL models and after one of these models has been deployed. They were conducted on an Ubuntu 22.04.4 LTS machine, with an Intel (R) Core (TM) i9-14900KF processor, featuring 32 logical CPUs and 2 NVIDIA GeForce RTX 4090 GPUs, each with 24 GB. The system architecture is x86_64, with the processor capable of running at a maximum frequency of 6.0 GHz. The evaluation process involved analyzing the captured data by submitting queries to the DLProv provenance database. Additionally, the overhead associated with provenance data capture was analyzed, comparing the execution times of DL workflows with and without provenance tracking. The results show that DLProv introduces minimal overhead, approximately 1.4%. This analysis allowed for an understanding of the performance trade-offs when integrating provenance services into DL workflows, highlighting both the benefits in terms of traceability and the computational cost involved.

### Simple DL model on MNIST dataset

In this first experiment, we implemented a code for training a DL architecture using PyTorch, which was then trained on the MNIST dataset (Lecun et al., 1998). The source code for these experiments was divided into functions and executed using Parsl *via* the *@python_app* decorator. These functions were organized as follows:

- **Data loading:** The dataset was loaded and preprocessed using load_data() task.
- **Model building:** DL architecture was defined in the build_model() task.
- **Model training:** The model was trained in the train_model() task on both GPUs over 50 and 100 epochs, using the Adam optimizer and a learning rate of 0.001.
- **Model evaluation:** The model was evaluated in evaluate_model() task on the test set to measure performance.

The DL training was performed using several hyperparameter configurations. These configurations included different learning rates and optimizers, each applied to preprocessed data with distinct sequences. Performance metrics such as accuracy, loss, and training time were captured throughout the training process. Such performance metrics can be analyzed during training through the execution of queries in MonetDB to answer questions such as “*What is the hyperparameter configuration used to train the model with the highest test accuracy?*”. By analyzing the results of different configurations, insights can be gained regarding the impact of hyperparameters on model performance.

A quantitative experiment was conducted to provide a comparative analysis of the cost of capturing provenance data using DLProv and MLflow during the training of a DL model. Specifically, the execution time of the workflow—from loading data to evaluating the trained model—was analyzed under three conditions: *(i)* without capturing provenance data (baseline), *(ii)* with provenance data capture, including relationships, using DLProv, and *(iii)* with metadata capture using MLflow.

#### Overview

The MNIST dataset, accessed through PyTorch (https://pytorch.org/vision/0.20/generated/torchvision.datasets.MNIST.html), consists of a training set with 60,000 examples of handwritten digits, covering the 10 digit classes, and a test set with 10,000 examples. For this experiment, we used a simple feed-forward neural network. The architecture consists of an input layer designed to handle 2D gray-scale images with a shape of 28 × 28 pixels, as found in the MNIST dataset. This is followed by a *Flatten* layer, a *Dense* layer, a *Dropout* layer, and a final *Dense* layer. We chose to train a simple DL architecture on the MNIST dataset because it is a well-known benchmark in the field of ML. The dataset offers an accessible starting point for those interested in exploring learning techniques and pattern recognition methods with real-world data, while requiring minimal effort in terms of preprocessing. Given that our goal is to show the analysis of DL workflows during model training, we found that MNIST provided an ideal context for showcasing these analyses.

#### Overhead analyses

**Figure 6:**
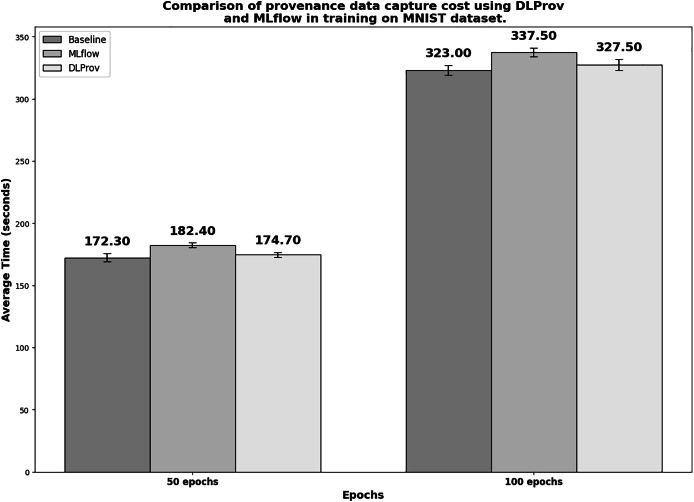
Execution time for 50 and 100 epochs—SimpleNN with MNIST.

Figure 6 shows the execution times in seconds for the simple DL model with MNIST. To obtain a reliable average, each experiment was executed 10 times. In the 50-epoch experiment with 2 GPUs, DLProv introduces minimal overhead compared to the Baseline, with an overhead of approximately 1.392%, which is quite small. In contrast, MLflow introduces a higher overhead of about 5.841%. These results indicate that DLProv has a relatively low impact on execution time when compared to the Baseline. The standard deviation for each tool (3.37 for Baseline, 1.96 for MLflow, and 1.95 for DLProv) shows that the times for DLProv and MLflow are fairly consistent, with a slightly higher variance for the Baseline. For the 100-epoch experiment with two GPUs, DLProv again shows a minimal overhead of about 1.393%, while MLflow’s overhead is approximately 4.489%. The standard deviations for this round (4.03 for baseline, 3.63 for MLflow, and 4.30 for DLProv) reflect a slightly higher variation in execution times compared to the 50-epoch experiment, but DLProv still remains stable.

#### Provenance query analyses

While training a simple DL model with the MNIST dataset, a data scientist’s goal is to identify the best candidate models and, ultimately, select the most suitable model for deployment. This process involves exploring hyperparameter configurations and applying different preprocessing techniques. To make informed decisions, it is important to analyze and compare the performance and characteristics of the trained models. DLProv facilitates this analysis by enabling queries on its DL Model Provenance database in MonetDB. To support these analyses, we submit queries Q1, Q2, Q3, Q4, and Q5, which provide insights into hyperparameters, performance metrics, and the data contributing to the models. Additionally, other queries from Table 2 can be leveraged during this stage to deepen the analysis.

Q1 focuses on identifying all trained models that used a specific learning rate, such as 0.001. As shown in Fig. 5, this can be achieved by joining the *itrain* and *otrainedmodel* classes. The result of this query in SQL provides details about the trained models, including an identifier, the model name, and the file path where the model is saved (*e.g*., /home/dbpina/parsl-exps/parsl-experiments/Resnet50/resnet50-trained.keras). This approach allows us to track and compare models based on their learning rate configuration.

Q2 can also leverage the join between the *itrain* and *otrainedmodel* tables. First, we filter the specific DL model by criteria such as learning rate (*e.g*., 0.001) and batch size (*e.g*., 32), or apply additional filters as needed. Then, by joining with the *otrain* table, we can calculate the average epoch processing time by aggregating the elapsed time for the corresponding training task, using the task identification associated with the DL model. This approach enables us to track and analyze the training performance, providing insights into the efficiency of the model training process.

Q3 follows a similar approach to Q2, but in this case, the goal is to identify the DL model with the highest training accuracy. To achieve this, we first calculate the average training accuracy for each model by averaging the accuracy values in the *otrain* table. We then find the maximum of these average accuracies. Using this maximum value, we can identify the specific DL model that achieved the highest training accuracy. This is done by performing a join between the *itrain* and *otrainedmodel* tables based on the *train_task_id*, allowing us to retrieve the corresponding hyperparameters and other relevant details for the model with the highest training accuracy. Listing 4 shows a snippet of this query.

**Listing 4:** Example of SQL syntax for querying Q3, illustrating the structure and components of a query designed to retrieve specific information about hyperparameters and accuracy.

1 SELECT im.*, om.avg_accuracy
2 FROM itrain im
3 JOIN (
4 SELECT train_task_id, AVG(accuracy) AS avg_accuracy
5 FROM otrain
6 GROUP BY train_task_id
7 HAVING AVG(accuracy) = (
8 SELECT MAX(avg_accuracy)
9 FROM (
10 SELECT AVG(accuracy) AS avg_accuracy
11 FROM otrain
12 GROUP BY train_task_id
13 ) AS subquery
14 )
15 ) om ON im.train_task_id = om.train_task_id;

Q4 also utilizes the *MAX* function in SQL, but unlike Q3, there is no need to compute an average since there is only one test accuracy value for each model. In this case, the goal is to identify the DL model with the highest test accuracy. By directly applying the *MAX* function to the test accuracy values in the *otest* table, we can find the model with the highest performance on the test set. We then perform a join with the *itrain* table, based on the *train_task_id*, to retrieve the corresponding hyperparameters and other details of the model that achieved the highest test accuracy.

Q5 delves further back into the derivation trace, focusing on the data that contributed to the highest test accuracy. Similar to Q4, which identifies the model with the highest test accuracy, Q5 examines the underlying data used in the training and evaluation of that model. The query begins by identifying the highest test accuracy stored in the *otest* table. Once this value is determined, a join with the *otrainedmodel* table is performed to locate the specific DL model that achieved this accuracy, based on *test_task_id*. Finally, the query links this model to the *odata_split* table, using *train_task_id*, which holds information about the datasets used during training and evaluation. This process enables us to trace back to the exact data that contributed to the training and testing of the model with the highest test accuracy, providing a comprehensive view of the data’s role in achieving this result.

### ResNet50 architecture on CIFAR-100 dataset

This second experiment involved training the ResNet50 architecture on the CIFAR-100 dataset (Krizhevsky & Hinton, 2009) using TensorFlow. Similar to the first experiment, this one followed the same process of organizing the source code into functions, which were applied *via* the *@python_app* decorator with Parsl, covering key tasks such as Data Loading, Model Building, Model Training, and Model Evaluation. In addition, a quantitative evaluation was performed to conduct a comparative analysis of the cost of capturing provenance data using DLProv and MLflow, mirroring the approach taken in the first experiment.

#### Overview

The CIFAR-100 dataset (https://keras.io/api/datasets/cifar100/), accessed through Keras’ built-in small datasets module (https://keras.io/api/datasets/cifar100/), consists of 100 classes, each containing 600 images. There are 500 training images and 100 testing images per class. These classes are grouped into 20 broader categories, or superclasses, such as animals, vehicles, household items, and natural scenes. Every image is annotated with both a fine label, indicating its specific class, and a coarse label, denoting its superclass.

We chose ResNet50 (He et al., 2016) for our second experiment, a deep residual network, due to its widespread adoption in the research community. By using ResNet50, we aimed to assess the provenance capture provided by DLProv in a more complex, state-of-the-art DL model in comparison to the simpler feed-forward neural network used in the first experiment. We used a model that builds on the ResNet-50 architecture available on the Keras webpage (https://keras.io/api/applications/resnet/#resnet50-function). The ResNet50 model is used as a feature extractor, with the top classification layer removed (‘include_top=False’). The model is initialized without pre-trained weights (‘weights=None’), meaning it starts training from scratch.

#### Overhead analyses

**Figure 7:**
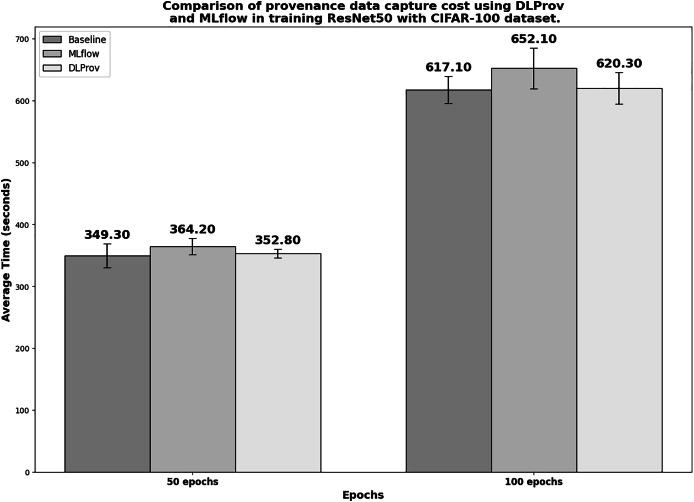
Execution time for 50 and 100 epochs—ResNet50 with CIFAR100.

Figure 7 shows the execution times in seconds for the ResNet50 with CIFAR-100. To obtain a reliable average, each experiment was executed 10 times. The 50-epoch experiment with two GPUs shows that DLProv adds minimal time compared to the Baseline, with an overhead of about 1.002%, and MLflow adds 4.265% of overhead. Interestingly, in the 100-epoch experiment with 2 GPUs, DLProv’s overhead is even lower, at approximately 0.519%, reinforcing its efficiency in capturing provenance data, while MLflow showed a higher overhead of about 5.672%.

During the initial runs of the 50-epoch experiment, the Baseline unexpectedly took longer than DLProv. This led us to investigate CPU usage, which showed fluctuations. In addition, many CPU cores were idle, causing inconsistent performance. To address this, we set CPU affinity to “block” in Parsl to assign adjacent cores to workers, and limited the experiments to use only 15 cores, which were the most active ones during training. After this change, the execution times became more consistent, and the Baseline ran as expected. Despite this, the Baseline’s runtime still exhibited a relatively high standard deviation for 50 epochs at 13.35 s, slightly higher than MLflow’s 13.13 s, and significantly higher than DLProv’s more stable runtime with a standard deviation of 7.13 s. For 100 epochs, the standard deviation increased across all cases: the Baseline showed 21.94 s, MLflow had the highest variation at 33.12 s, and DLProv showed relatively better stability than MLflow with a standard deviation of 25.45 s.

#### Provenance query analyses

For the ResNet50 training with CIFAR-100, consider a scenario where we trained different model configurations and obtained model results. We have analyzed and selected a DL model among the candidates to be deployed. Following the completion of the experiment, with the selection of the DL model to be deployed, a provenance document was generated with the steps that led to it. This graph includes information such as the hyperparameters used, the preprocessing steps applied, and details of the DL model architecture. By capturing the full provenance, the graph is a traceable record of the model development process, enhancing transparency and reproducibility in a production environment.

**Figure 8:**
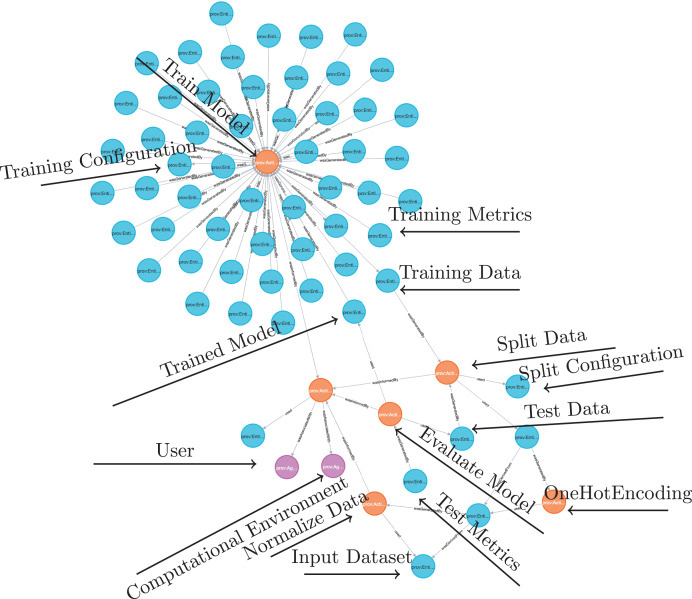
Provenance graph in Neo4j showing the deployed DL model’s derivation trace.

The provenance document, available in PROV-N or JSON, is generated from the provenance data stored in MonetDB immediately after the DL model is selected for deployment. Once generated, the document is packaged with the DL model. Queries can then be submitted by importing the provenance document into Neo4j, leveraging the functionality provided by DLProv, or using a dedicated solution such as the PROV Database Connector (https://github.com/DLR-SC/prov-db-connector). This setup allows for the querying of the provenance graph. Figure 8 shows the provenance graph for the deployed DL model in Neo4j. The blue circles represent entities, the orange circles denote activities, and the purple circles correspond to agents.

For this deployed DL model, we submit queries Q7, Q8, Q9, Q10, Q11, and Q12. It is important to note that some of these queries can also be performed during the generation of DL models and their training. In such cases, they can be answered using MonetDB.

Query Q7 seeks to retrieve the hyperparameters used in the deployed DL model, identified as *M4*. During the provenance document’s creation, DLProv assigns a unique identifier to each model; in this case, it is *dlprov:99ad4e82-17b1-49f1-86ad-b52cd7dcab6e*. However, we will avoid using this identifier in the article for better readability. The response to this query involves the entity *oTrainedModel*, which captures the details of M4, the DL model resulting from training. This entity is linked to the activity *Train* through the *wasGeneratedBy* relationship and is associated with the hyperparameters entity *iTrain via* the *used* relationship. The hyperparameters recorded include the optimizer Adam, learning rate 0.001, 100 epochs, 179 layers, and a batch size of 32.

Query Q8 focuses on identifying the computational environment used to train a DL model. Similar to the approach for Q7, this query retrieves information from the provenance data associated with the activity *Train*. However, instead of targeting the hyperparameters, the emphasis is on the computational environment *used* during the training of model M4. The response reveals that the computational setup included the operating system Ubuntu 22.04.4 LTS, an i9-14900KF processor, 15 CPUs, and 2 NVIDIA GeForce RTX 4090 GPUs.

Query Q9 seeks to identify the Agent that *wasAssociatedWith* the activity *Train*. Similar to the approaches for queries Q7 and Q8, this query focuses specifically on the agent responsible for the training process. The response involves the entity *oTrainedModel*, which is connected to the activity *Train via* the *wasGeneratedBy* relationship. This activity, in turn, is linked to the agent *User* through the *wasAssociatedWith* relationship. The agent’s information includes the name and may also include additional details, such as an email address, providing accountability and traceability for the model training process.

Query Q10 investigates the entire process that led to the creation of model M4, tracing all activities and entities involved in producing this deployed DL model. The answer to this query relies on navigating the provenance graph to identify the sequence of activities, their associated agents, and the entities they *used* or *generated*. Starting from the entity *oTest*, which contains the set of metrics that *wasGeneratedBy* the activity *Test*, which *used* the test set to evaluate the *oTrainedModel*, which represents model M4. We traverse its *wasGeneratedBy* relationship to the activity *Train* that *used* a training set. From here, we can explore all connected inputs and outputs, such as the preprocessed datasets, hyperparameters, computational environment, and intermediate steps, such as the activity *SplitData*. Additionally, it captures the feature transformations applied to the preprocessed dataset and links back to the original input dataset. This traversal provides a complete derivation trace, detailing how each element contributed to the model’s creation.

Query Q11 retrieves the dataset used to train the deployed DL model. Using the DLProv provenance graph, we can trace the derivation path starting from the trained model, represented by the entity *oTrainedModel*, which *wasGeneratedBy* the activity *Train*. This activity *used* a specific dataset, identified as *oTrainSet*. In this case, the dataset comprises images stored in the file located at the path *temp_cifar100/train.npz*.

Query Q12 focuses on identifying the preprocessing operations applied to the input data, represented by the entity *iInputDataset*. With the integration capabilities provided by the DLProv suite, which connects different steps in the DL workflow, this query can be executed using Cypher. The result offers a derivation trace starting from the trained model entity *oTrainedModel*, traversing back to the preprocessing activities associated with *iInputDataset*. For instance, in the training of ResNet50 on the CIFAR-100 dataset, two preprocessing operations were applied. The resulting preprocessed dataset *oOneHotDataset wasDerivedFrom* a *OneHotEncoding* activity, which, in turn, *used* an entity *oNormalizeData* that *wasDerivedFrom* a *NormalizeData* activity. The latter directly *used* the original input data *iInputDataset*.

Although the provenance capture for these experiments was conducted using different DL frameworks, PyTorch and TensorFlow, it is important to emphasize that the provenance data representation remains consistent. This consistency facilitates interoperability, enabling analysis and comparison of provenance information across different DL frameworks.

Some of the queries discussed, whether with SQL or Cypher, cannot be answered using frameworks, such as MLflow and MLflow2PROV, that fail to capture relationships that allow this type of analysis, and even the level of detail. This limitation arises because MLflow focuses primarily on high-level experiment tracking, such as logging parameters, metrics, and artifacts, without integrating detailed provenance information about the data transformations, computational environments, or derivation traces of entities. Similarly, MLflow2PROV extends MLflow by mapping its tracking information to PROV, but it relies on the scope of MLflow’s original data. Queries like Q5, which require tracing data contributions through a detailed derivation trace, or Q10, which investigates the derivation process of a DL model, require a richer provenance graph that captures all steps and entities in the DL workflow. These queries highlight the need for more comprehensive provenance solutions, such as DLProv, which explicitly integrates fine-grained data and workflow provenance into its model.

### Handwritten transcription workflow execution

The last experiment focused on the inference step for transcribing handwritten Portuguese texts (https://github.com/MeLLL-UFF/handwriting-transcription-ptbr) (da Silva et al., 2023). Unlike the previous workflows, which were centered around model training, this experiment involved inference, where a trained model is used to make predictions. To accommodate this shift, the DLProv suite’s provenance model was extended to capture the activities and entities specific to the inference workflow. In this workflow, we added a comparison step, where the inferred text is compared to the ground truth, which was included primarily to generate performance metrics for provenance analysis. Although metrics are typically associated with training and evaluation rather than inference, incorporating them here allows us to show how DLProv can capture and query relationships between inputs, outputs, and performance in an inference scenario.

#### Overview

**Figure 9:**
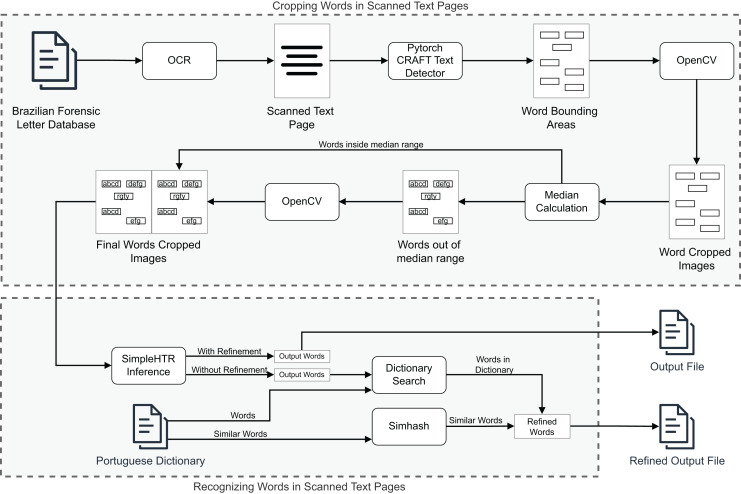
Workflow for transcribing handwritten Portuguese. Adapted from da Silva et al. (2023).

In this case study, the handwritten transcription process involves several steps: reading an input image, detecting regions within the image that contain textual information, recognizing the text within each region, and transcribing it into a file. These transcriptions are then combined to reconstruct the full text. To evaluate the processes described, we used the Brazilian Forensic Letter Database (Freitas et al., 2008), consisting of 945 image samples of the same text, written by 315 authors, with each author having written three texts by hand. The pages containing the text are scanned using Optical Character Recognition (OCR) in a gray scale, generating the images contained in the database. From these scanned text pages, word bounding areas are identified using the CRAFT text detector. These images undergo an initial processing to obtain only the words contained in each text. To do this, a technique is applied for detecting word areas based on the identification of characters in images and their respective affinity regions, combined to form the region that corresponds to a word. From the demarcation of these spaces, an additional process extracts the words, resulting in a new set of images, each containing one word. OpenCV is used to crop the word images, which are then filtered by computing a median word size range. In this set, certain regions that encompass more than one word were detected, resulting in an image containing both. Because of this, a second process is performed to separate these words into distinct images. In other cases, due to aspects inherent to the writing style of the text, certain regions of words are not identified, and a technique is proposed to change aspects of the image in an attempt to make the information contained therein more expressive, approaching common characteristics of images in which text identification is possible. The final set of word images goes through a SimpleHTR inference model that outputs recognized words. These outputs are optionally refined through a process involving dictionary search using a Portuguese lexicon and Simhash. The output and its refined version are saved in separate output files. To validate the recognized words, we compared the inferred text to the original text and computed the accuracy of this inference. Figure 9 shows the activities in this workflow.

#### Provenance query analyses

**Figure 10:**
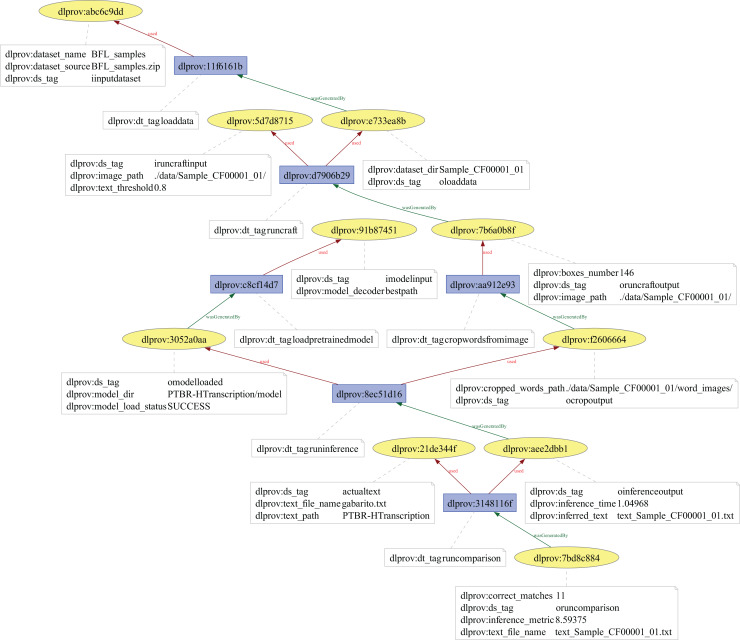
Fragment of the W3C PROV provenance graph showing the derivation trace of the inference of a sample in the workflow for transcribing handwritten Portuguese.

Figure 10 shows a fragment of the provenance graph generated from the inference of a single sample. For clarity, certain nodes have been omitted, and the identifiers shortened to enhance readability. This provenance graph traces the handwritten transcription workflow, starting with loading the dataset (*LoadData*) and a pretrained model (*LoadPretrainedModel*), followed by crafting word regions (*RunCraft*), cropping images (*CropWordsFromImage*), and performing inference (*RunInference*). The last activity in the provenance graph is a comparison (*RunComparison*) between the inferred and the original text, capturing metrics such as accuracy. The activities *RunCraft* and *CropWordsFromImage* are part of the data preparation step in the DL workflow. In this experiment, we show not only the capability to extend the data to be captured but also DLProv’s ability to capture preprocessing operations while seamlessly integrating with DL model training or inference processes.

To evaluate the provenance captured by DLProv in this experiment, we can adapt the provenance queries from Table 2 to fit this context. For instance, while Q6 is typically used during DL model development, it can be modified for the inference step to analyze relevant metrics, identified as *inference_metric* in entity labeled as *“dlprov:ds_tag”:“oruncomparison”*, which serves as a key evaluation measure here. Since we captured accuracy instead of loss, we can adapt the query to *Given a set of samples of the same text, what are the sample, threshold, and model decoder values and the evaluation measure values associated with the inference that has the highest accuracy?*. This query is performed over multiple executions, not only on the one presented in Fig. 10. To answer this, we first need to find the highest accuracy (*inference_metric*) and then trace the entity containing this highest value, which *wasGeneratedBy* the activity *RunComparison*. From there, we trace back to the entities *oRunCraftOutput* containing the sample, *iModelInput* containing the decoder, and *iRunCraftInput* containing the threshold, which together led to this accuracy.

This query can also be adapted to answer *Given sample Sample_CF00001_01, what are the values of the threshold used and the accuracy achieved?*, maintaining the same structure as the original query by relating the input data (the sample), the parameter (the threshold), and the metric (the accuracy). The answer to this query follows a derivation process similar to that of the previous graph analysis. It reveals that a threshold of 0.4 resulted in an accuracy of 6.25%, while a threshold of 0.8 achieved an accuracy of 8.59%.

In this experiment, without a provenance represented as a graph document that can be queried, there is no information about the trained model being used for inference. We do not know the type of data it was trained on, the hyperparameter values used, or how the data was prepared. In the query example presented, we used different threshold values, which led to a variation in the accuracy. This raises the question: could this difference be caused by a mismatch between the inference process and the original training process, of which we have no knowledge? This lack of transparency highlights the need for a provenance document to accompany the DL model, as shown in the ResNet50 experiment. Such documentation would provide traceability into the training process, including details on data, hyperparameters, and data preparation, allowing for a better understanding of discrepancies during inference.

## Conclusion and future work

Supporting traceability of DL workflows is important for enabling thorough analysis during the generation and selection of DL models. This helps improve decision-making by helping data scientists identify which aspects of the workflow should be tuned and which model should ultimately be deployed. Moreover, traceability plays a key role in ensuring reproducibility and building trust in deployed DL models. By capturing detailed information about the DL model, including the data and hyperparameters used, the preparation steps, and other relevant data, traceability ensures that the process is transparent and that DL model users can trust the model’s performance and reliability.

In this article, we introduced the DLProv suite, a solution designed to allow traceability by capturing provenance across the steps of DL workflows and representing key relationships. The DLProv suite includes specializations for Keras, PINNs, and the DL life cycle. Our contributions comprise *(i)* an extensible provenance model capable of representing activities, agents, and entities specific to DL tasks, such as data preparation, model training, and evaluation; *(ii)* integrated provenance capture mechanisms, exemplified by KerasProv, which enables the automatic capturing of hyperparameters, configurations, and metrics, and PINNProv, which captures specific components of PINNs; *(iii)* the generation of provenance documents that facilitate detailed queries, enabling traceability and analysis, and promote trust and reproducibility of deployed DL models; and *(iv)* the adoption of W3C PROV as a standard representation, which ensures interoperability across different environments.

Our experiments showed DLProv’s ability to capture and analyze provenance across multiple DL scenarios. For example, we showed how provenance queries can relate input data, hyperparameters, and evaluation metrics, enabling detailed insights into the impact of preprocessing and configuration decisions on model performance. Additionally, we validated DLProv’s capacity to adapt to different contexts, such as tracking inference accuracy and thresholds for specific samples, highlighting its flexibility in addressing a range of provenance-related challenges.

For future work, we plan to explore provenance capture for the execution of DL workflows at different levels of granularity and bundle them together with their associated objects, enabling a more detailed representation of the workflow. By capturing provenance at multiple levels, we can provide greater flexibility for data scientists to analyze workflows at a high level or drill down into specific details as needed. Moreover, future work involves addressing the challenge of capturing provenance in continuum computing environments. Building on our experience with parallel environments using Parsl and edge computing (Rosendo et al., 2023), we plan to develop methods for capturing and managing provenance data in dynamic, real-time workflows.

Beyond exploring DLProv, it is important to promote the adoption of provenance solutions in the DL research and development community. Many DL researchers and developers are unaware of these tools or view them as an unnecessary burden. To address this, future work should also focus on enhancing usability, integrating provenance capture seamlessly into DL workflows, and showing its benefits in model development and selection, analyses, transparency, and reproducibility. Engaging with the ML community through case studies, intuitive interfaces, and integration with popular frameworks can help establish provenance as a standard practice rather than an optional overhead.

## Additional Information and Declarations

### Data Availability

The following information was supplied regarding data availability:

The MNIST dataset is available at https://pytorch.org/vision/0.20/generated/torchvision.datasets.MNIST.html.

The CIFAR-100 dataset is available at https://keras.io/api/datasets/cifar100.

The DLProv code is available at GitHub and Zenodo:

- https://github.com/dbpina/dlprov.

- Débora Pina. (2025). DLProv (v1.0.0). Zenodo. https://doi.org/10.5281/zenodo.15272133.
